# A Predictive Model for Corticosteroid Response in Individual Patients with MS Relapses

**DOI:** 10.1371/journal.pone.0120829

**Published:** 2015-03-18

**Authors:** Martin Rakusa, Stefan J. Cano, Bernadette Porter, Afsane Riazi, Alan J. Thompson, Jeremy Chataway, Todd A. Hardy

**Affiliations:** 1 Queen Square Multiple Sclerosis Centre, Department of Neuroinflammation, UCL Institute of Neurology, University College London and National Hospital for Neurology and Neurosurgery, University College London Hospitals NHS Foundation Trust, London, United Kingdom; 2 Clinical Neurology Research Group, Room N16 ITTC Building, Plymouth University Peninsula Schools of Medicine and Dentistry, Tamar Science Park, Davy Road, Plymouth, United Kingdom; 3 University College London, Institute of Neurology, Dept of Brain Repair and Rehabilitation, London, United Kingdom; 4 Department of Psychology, Royal Holloway, University of London, Surrey, United Kingdom; 5 MS Clinic, Brain & Mind Research Institute, University of Sydney, Sydney, Australia; 6 Neuroimmunology Clinic, Concord Repatriation General Hospital, Sydney, Australia; University of Düsseldorf, GERMANY

## Abstract

**Objectives:**

To derive a simple predictive model to guide the use of corticosteroids in patients with relapsing remitting MS suffering an acute relapse.

**Materials and Methods:**

We analysed individual patient randomised controlled trial data (n=98) using a binary logistic regression model based on age, gender, baseline disability scores [physician-observed: expanded disability status scale (EDSS) and patient reported: multiple sclerosis impact scale 29 (MSIS-29)], and the time intervals between symptom onset or referral and treatment.

**Results:**

Based on two a priori selected cut-off points (improvement in EDSS ≥ 0.5 and ≥ 1.0), we found that variables which predicted better response to corticosteroids after 6 weeks were younger age and lower MSIS-29 physical score at the time of relapse (model fit 71.2% - 73.1%).

**Conclusions:**

This pilot study suggests two clinical variables which may predict the majority of the response to corticosteroid treatment in patients undergoing an MS relapse. The study is limited in being able to clearly distinguish factors associated with treatment response or spontaneous recovery and needs to be replicated in a larger prospective study.

## Introduction

Relapsing-remitting multiple sclerosis (RRMS) is characterised by recurrent attacks of neurological symptoms reflecting underlying central inflammatory demyelination [1]. MS relapses vary in terms of their frequency, location, severity and outcome.

More than 40% of patients who go untreated with corticosteroids are left with residual deficits [expanded disability status scale (EDSS) ≥ 0.5] an average of 9 weeks after a relapse [2]. Corticosteroids hasten recovery from relapses and these are frequently offered to patients in the acute setting [3–5]. For every 1000 MS relapse patients treated with corticosteroids, 247 more will improve compared to placebo [6,7]. Corticosteroids are believed to exert their effect by a number of mechanisms including reducing pro-inflammatory cytokines and modulating B and T lymphocytes [8].

Despite these studies, questions remain as to the underlying clinical variables that predict why some patients with RRMS improve with treatment, whilst others respond poorly. To date, relatively little has been reported about the potential factors that predict response to corticosteroids, even though their clinical use is widespread. The aim of this study was to develop a simple predictive clinical model, using patient and clinician reported outcomes, to determine for an individual patient suffering from an MS relapse, whether or not corticosteroids are likely to be beneficial.

## Materials and Methods

### Patients

Data were collected from patients originally recruited into a randomised controlled trial comparing intravenous methylprednisolone (IVMP) 1g daily for three days for acute MS relapses given either in an ambulatory out-patient setting or at home [9]. All patients were 18 years or older and reviewed in the weekly MS relapse clinic at the National Hospital for Neurology and Neurosurgery, London, UK. Patients were diagnosed with MS according to 2001 McDonald criteria [10]. Only patients with RRMS, defined as a lack of disability progression between relapses, were included in the study.

Acute MS relapses were defined as neurological episodes of more than 24 hours and less than 4 weeks duration as defined by Poser [11]. Patients were included if, according to these criteria, they had a moderately severe relapse defined as an episode causing functional impairment. They were excluded if they had evidence of intercurrent infection or previous adverse side effects after steroid use. Patients with mild relapses, that is, defined by the treating physician not to have a significant effect on function e.g. numbness due to a pure sensory relapse or mild visual blurring from optic neuritis, were also excluded; as were patients with relapses severe enough to require inpatient hospitalisation.

Characteristics including the EDSS and the Multiple Sclerosis Impact Scale-29 [v1.0] were collected at baseline and re-assessed at 6 weeks following treatment. The EDSS is a widely used clinician-assessed outcome measure of disability with a range of severity from 0 to 10 in which 1 represents no disability or minimal signs in one functional system and 10 represents death due to MS [12]. The MSIS-29 v1.0 is a patient-reported outcome (PRO) measure consisting of 29 self-report questions rating the physical and psychological impact of MS on patient’s lives over the preceding two weeks on a scale from 1 to 5 (i.e. 1 representing no limitation in ability and 5 representing extreme limitation in ability). There are two subscale scores, physical (range 20–100) and psychological (9–35). The MSIS-29 has been shown previously to be a clinically useful and valid measure of disability in persons with multiple sclerosis [13].

Ethics approval was not required for the current study as it was a retrospective audit of data that had already been collected during a larger study which had approval from the Ethics Committee of University College London Hospital, UK. Written informed consent was obtained from all patients.

### Statistical Analysis

We selected key patient variables of: age, gender, baseline EDSS, MSIS-29 physical and psychological subscales, number of exacerbations in the last two years, number of corticosteroid treatments, disease duration since diagnosis of MS, years since index symptom of demyelination and time interval between symptom onset or referral and treatment, to define the model. The index symptom of demyelination was defined as the first symptom a patient experienced which would be compatible with a relapse, whether it occurred as a clinically isolated syndrome or was appreciated retrospectively at the time of MS diagnosis. A pre-relapse EDSS was not included as it could not be reliably determined from the patient’s clinical notes in all cases. As in other studies assessing the relationship between multiple variables in MS patients, the analysis used binary logistic regression (the ENTER method) to explore which variables influenced treatment outcome [14]. We used a priori thresholds of improvement of EDSS ≥ 0.5 and ≥ 1.0, with the chance of successful treatment calculated using the standard model:

$$chanceofsuccessfultreatment=\frac{1}{1+e^{-z}}$$

where z = B + B_1_variable 1 + B_2_variable 2 + … B_x_variable x where B is the co-efficient of the predictor variables in the logistic regression; e is base of the natural logarithm. Baseline characteristics between male and female patients and EDSS and MSIS-29 scores at relapse, between treatment responders and non-responders were assessed for differences using one way ANOVA. Non parametric correlation (Spearman’s rho) was calculated between baseline EDSS, baseline MSIS-29 physical and MSIS-29 psychological. Correlation was also calculated for the difference between EDSS and MSIS-29 at baseline and 6 weeks. Chi square analyses were used to test the significance of the logistic model. *P*-values of ≤ 0.05 were considered significant.

## Results

**Fig 1:**
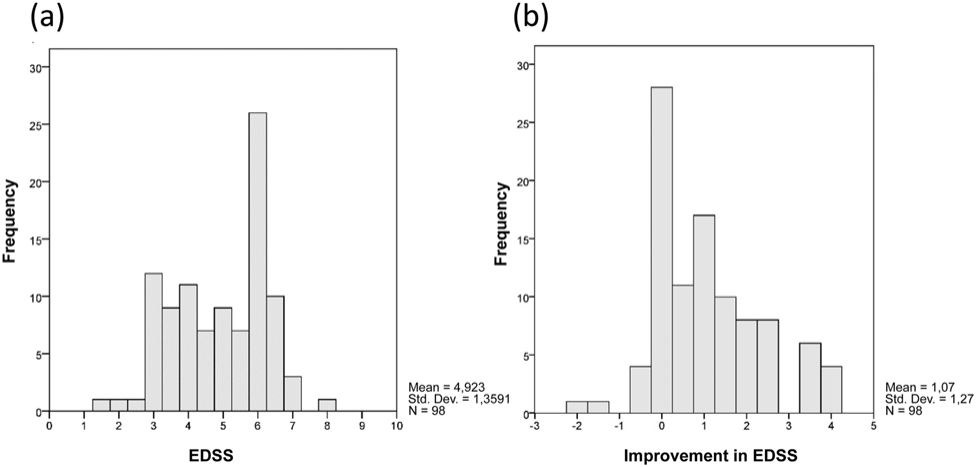
Distribution of baseline EDSS at the time of MS relapse (a) and improvement in EDSS after treatment with intravenous methylprednisolone (b).

The patient characteristics are displayed in Table 1. 98 of 113 patients with RRMS from the original study had a complete data set and were included in this study. 75 were female and the mean age was 39 years (see Table 1). The average time from relapse onset to treatment was 17 days (SD ± 7) and from referral to treatment was 5 days (SD ± 3). The mean baseline EDSS score following an attack was 4.9 which improved on average by 1.1 when re-measured at 6 weeks (Table 1, Fig. 1). Baseline EDSS and MSIS-29 physical correlated well (rho 0.5, *p* < 0.01) as well as baseline MSIS-29 physical and MSIS-29 psychological (rho 0.6, *p* < 0.01). There was also a positive correlation between the difference in EDSS and MSIS-29 physical measured at 6 weeks (rho 0.39, *p* < 0.01).

**Table 1:** Cohort data and variables used to define the model.

	Women (n = 75)		Men (n = 23)		Total (n = 98)	
	Mean	SD	Mean	SD	Mean	SD
Age (years)	38.7	8.7	38.3	7.7	38.6	8.5
Baseline EDSS	5.0	1.3	4.8	1.5	4.9	1.4
Baseline MSIS-29 physical	68.1	19.0	68.0	22.1	68.1	19.6
Baseline MSIS-29 psychological	30.3	11.0	30.2	13.1	30.3	11.4
Relapses in the last 2 years	2.2	1.7	1.7	1.1	2.1	1.6
IVMP treatments in the last 2 years	0.9	1.3	0.7	0.8	0.8	1.2
IVMP and oral corticosteroids in the last 2 year	1.4	1.7	1.3	1.2	1.3	1.6
Years since index symptom	9.2	7.3	10.0	8.4	9.4	7.5
Interval between symptom onset andtreatment (days)	16.7	7.2	18.4	6.5	17.1	7.1
Interval between referral andtreatment (days)	5.0	2.8	6.6	3.4	5.4	3.0
Improvement in EDSS at 6 wks	1.1	1.3	1.0	1.3	1.1	1.3
Improvement in MSIS-29 physical at 6 wks	18.6	17.3	19.3	15.0	16.6	16.8
Improvement in MSIS-29 psychological at 6 wks	12.6	7.4	11.9	6.9	14.9	7.3

EDSS = expanded disability status scale, MSIS = multiple sclerosis impact scale, IVMP = intravenous methylprednisolone, sd = standard deviation, wks = weeks.

34 patients (35%) had no improvement in EDSS (≤0.5) or worsened following corticosteroids (Fig. 1b). There was no difference in the degree of baseline impairment (either EDSS or MSIS-29 physical) in younger patients (defined as ≤ the median age of 38 years) compared to older patients (> 38 years). Younger patients with shorter time since their index symptom of demyelination were more likely to respond to treatment to with corticosteroids than those patients who were older with a longer course (EDSS ≥ 0.5; *p* < 0.05; Table 2). For EDSS ≥ 1.0, younger patients remained more likely to respond to therapy (*p* < 0.05) but years since index symptom was not a significant factor (Table 3).

**Table 2:** Demographic variables between corticosteroid treatment responders and non-responders (EDSS change ≥0.5).

	Non-responder (n = 34)				Responder (n = 64)			
	Mean	SD	95% Confidence Interval for Mean		Mean	SD	95% Confidence Interval for Mean	
			Lower Bound	Upper Bound			Lower Bound	Upper Bound
Age*	41.0	9.3	37.7	44.2	37.4	7.8	35.4	39.3
Years since index symptom*	11.5	8.2	8.6	14.4	8.2	7.0	6.5	10.0
Baseline EDSS	5.1	1.3	4.7	5.6	4.8	1.4	4.5	5.1
EDSS at 6 weeks	5.3	1.3	4.8	5.8	3.1	1.6	2.7	3.5
Improvement in EDSS at 6 weeks	-0.2	0.4	-0.3	0.0	1.7	1.1	1.5	2.0
Baseline MSIS-29_physical_	75.9	15.6	70.4	81.3	63.9	20.4	58.8	69.0
MSIS-29_physical_ at 6 weeks	66.0	19.4	59.2	72.8	43.5	17.2	39.2	47.8
MSIS-29_psychological_ at 6 weeks	26.9	9.7	23.5	30.3	20.9	9.6	18.5	23.3
Improvement in MSIS-29_physical_ at 6 weeks	9.8	19.8	2.9	16.7	20.4	17.0	16.2	24.7

Only variables that were statistically significant are included in the table with the exception of baseline EDSS which was not significant.

*-p <0.05; all other variables p <0.01.

**Table 3:** Demographic variables between corticosteroid treatment responders and non-responders (EDSS change ≥1.0).

	Non-responder (n = 45)				Responders (n = 53)			
	Mean	SD	95% Confidence Interval for Mean		Mean	SD.	95% Confidence Interval for Mean	
			Lower Bound	Upper Bound			Lower Bound	Upper Bound
Age	40.8	9.0	38.1	43.5	36.8	7.6	34.7	38.9
EDSS at 6 weeks	5.1	1.4	4.6	5.5	2.8	1.5	2.4	3.2
Baseline EDSS	5.1	1.4	4.6	5.5	4.8	1.3	4.4	5.2
Improvement in EDSS at 6 weeks	0.0	0.5	-0.1	0.1	2.0	1.0	1.7	2.3
MSIS-29_physical_ at 6 weeks	62.1	20.6	55.9	68.3	42.2	16.5	37.6	46.7
MSIS-29_psychological_ at 6 weeks*	25.2	9.7	22.3	28.2	21.1	9.9	18.3	23.8
Improvement in MSIS-29_physical_ at 6 weeks	9.6	17.7	4.3	15.0	22.8	17.3	18.0	27.6
Improvement in MSIS-29_psychological_ at 6 weeks	4.2	9.3	1.4	7.0	9.9	14.4	5.9	13.9

Only variables that were statistically significant are included in the table with the exception of baseline EDSS which was not significant.

*-p <0.05; all other variables p <0.01.

After we performed logistic regression, variables which predicted a significant response to corticosteroids at the two threshold levels were: age and MSIS-29 physical. A better response to corticosteroids was seen for younger patients and those with a lower MSIS-29 physical score. Percentages from the contingency table were 73.1% and 71.2% for EDSS ≥ 0.5 and EDSS ≥ 1.0 respectively indicating that our model fitted well. The other variables did not predict steroid responsiveness.

**Fig 2:**
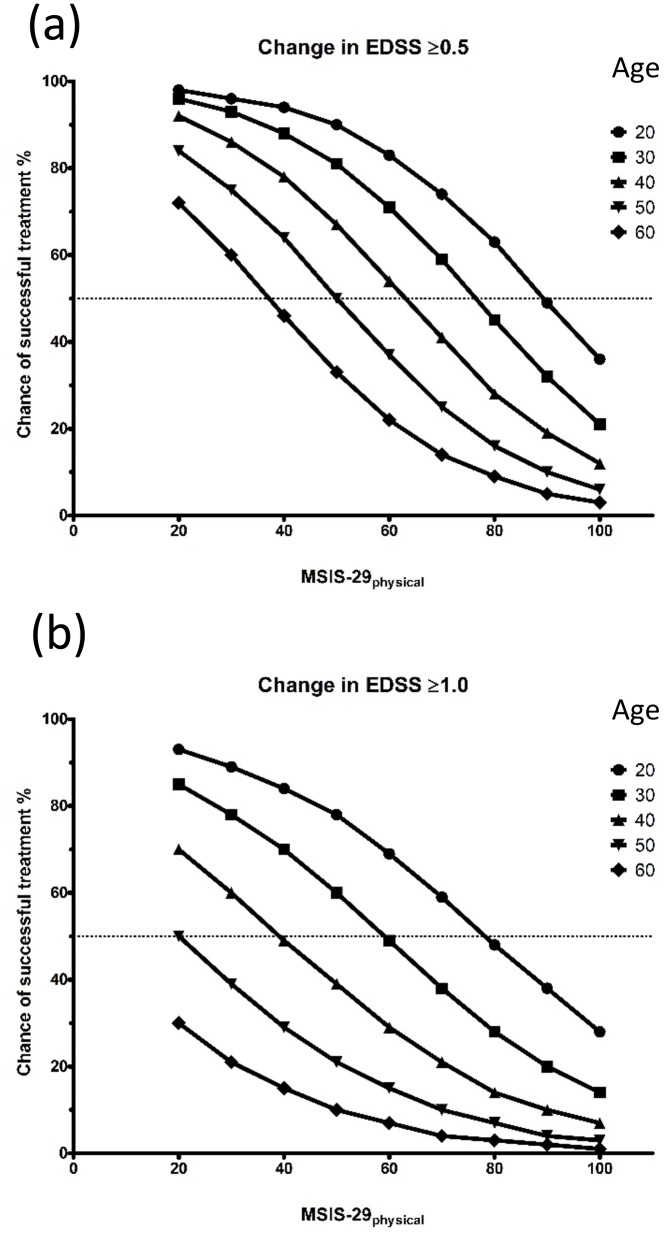
Chance of successful treatment at different patient ages and MSIS-29 physical scores for (a) improvement in EDSS ≥ 0.5 and (b) improvement in EDSS ≥1.0.

*Example*: The chance of successful treatment (EDSS ≥ 0.5) for a patient age 30 years with baseline MSIS-29 physical of 30 points can be estimated graphically in Fig. 2a by interpolating these variables to derive the chance of successful treatment of 95%. For the same patient the chance of improvement of EDSS ≥ 1.0 is 78% (Fig. 2b). If the patient was 50 years old, with the same baseline MSIS-29 physical, the corresponding chances would be 75% and 40% respectively.

## Discussion

In this study improvement in EDSS was used as a monitor of response to corticosteroids. While the EDSS has well-described limitations [15], it has the distinct advantage of being widely used and accepted [15], and setting the boundaries at ≥0.5 and ≥1.0 is a pragmatic way of allowing us to compute an anticipated response to treatment for relevant variables. From our modelling, the most important factor appeared to be age, with younger patients responding more favourably to treatment. Attack severity is also important with patients suffering from less severe attacks (i.e. lower MSIS-29 physical score) likely to benefit the most from treatment (Fig. 2). The study is novel in including a Patient Reported Outcome (PRO) tool, in addition to EDSS, as an outcome predictor of relapse disability and it may be that such a tool is a more sensitive mechanism for detecting the impact of relapses and their response to treatment than EDSS alone.

Published work on clinical factors predicting steroid-responsiveness is relatively limited and has not been extensively modelled (Table 4). The largest previous pure RRMS study retrospectively analysed data from 174 patients with relapsing remitting MS with relapse severity and residual disability by EDSS assessed at 1 month and 1 year following treatment with IVMP [16]. Severity of the relapse was defined as the difference between the peak relapse EDSS and the pre-relapse baseline EDSS. Patients in this study were slightly younger than in ours (mean age at relapse 35.6 years vs 38.6 years). Binary logistic regression found that severe relapses predicted residual disability at 4 weeks and 12 months after relapse. Younger patients (< 30 years) were likely to suffer more severe relapses but age was not a predictive variable in determining recovery from relapse at 1 year [16].

**Table 4:** Overview of prognostic factors determining a poorer relapse recovery.

	n	Population	Greater relapse severity	Increased age	Decreased age	Longer duration	Poor recovery from prior relapse	Relapse site	Polysite relapse	Polysymptom relapse	Efferent vs afferent relapse symptoms
Nos *et al*., 2004 [17]	51	CIS or RRMS	+	-	-	-		-			
West *et al*., 2006 [19]	186	CIS	+	-	-	-		-	-	-	
Leone *et al*., 2008 [18]	72	CIS or RRMS	+	+	-	+		-	+	+	+
Mowry *et al*., 2009 [20]	330	CIS or RRMS	+	-	-	-	+	spinal cord			
Vercellino *et al*., 2009 [16]	174	RRMS	+	-	-	-			-	-	
Hirst *et al*., 2012 [21]	144	RRMS	+	-	+	-					
Rakusa *et al*.	98	RRMS	+	+	-	-					

CIS = clinically isolated syndrome, RRMS = relapsing remitting multiple sclerosis. + means that a poorer outcome is associated with the variable,—means tested for and not significant, blank means not tested.

Another study examined factors predicting relapse recovery following corticosteroids among 51 patients (n = 54 attacks) with a clinically isolated syndrome (CIS; n = 10) or RRMS (n = 44) who were divided into three groups according to time to treatment from onset of symptoms (<4 weeks, 4–8 weeks and >8 weeks) [17]. The severity of the relapse was calculated using the change in EDSS from pre-relapse to attack state, with mild being <1.0, moderate 1 to 2.5 and severe ≥3.0 points. Linear modelling also demonstrated more severe attacks at one month as being the only predictor of response. This study differed significantly from ours in the inclusion of CIS patients, more severe relapses, smaller sample size and earlier treatment.

Other studies have looked at factors which predict short and longer term recovery following MS relapses in mixed cohorts of steroid treated and untreated patients [18–21]. A univariate model in 72 patients found that independent predictors of incomplete recovery were relapses of greater severity, patients with older age, efferent rather than afferent symptoms, relapses with more than one symptom, and longer relapse duration [18]. In this study, the single strongest predictor of incomplete recovery on multivariate analysis was relapse severity followed by total relapse duration and then age. In 186 patients suffering their first ever clinical demyelinating event, among whom 40% were treated with corticosteroids, univariate logistic regression showed greater severity, polyregional and polysymptomatic onset were associated with poorer recovery [19]. A study of 330 patients with CIS or newly diagnosed MS also found that event severity and degree of recovery from a previous event was an important predictor of relapse recovery with increased age weakly associated with poorer recovery [20]. Spinal cord onset also resulted in more significant longer term sequelae. Binary logistic regression applied to 144 patients undergoing acute MS relapse revealed that younger age and severity of relapse were predictive of severe residual disability [21].

The baseline disability of the patients included in our study at the time of relapse is higher (Mean EDSS 4.9) than in the study of Nos which included CIS patients (Median EDSS 3.0) but seemingly lower than in the study of Vercellino although peak relapse EDSS is not explicitly reported in the latter study [16,17]. It is certainly less than other studies looking at relapsing forms of MS [21, 22]. The level of disability in our study may reflect the fact that patients with CIS and mild relapses were excluded as patients with mild relapses are commonly managed conservatively [23].

The finding that older patients undergoing relapses are less likely to respond to treatment is noteworthy, and is illustrated by the worked example above where the chance of EDSS improvements of ≥ 0.5 and ≥ 1.0 reduced by 20% and 30% respectively for a 20 year increase in age. It seems that this can only be partly attributed to having the disease for longer, as the time from index symptom only predicted poorer response to corticosteroid for EDSS ≥ 0.5 but not for EDSS ≥ 1.0. However, in older patients with MS, acute lesions enhance less on MRI than in their younger counterparts [24] and so it may be that an increase in age causes subtle differences in lesion pathobiology which also makes them less responsive to corticosteroids. This is not to say that a relatively older age would predicate against corticosteroid use; relapses are damaging both physically and psychologically [25] and older patients may be more vulnerable due to comorbidities, financial limitations, fewer social opportunities and the need for more assistance [26]. However, the chance of success may be lower, and the potential risks versus benefits of treatment need to be carefully considered.

Our pilot study has limitations. The original trial from which this prospective data were taken, looked at patients’ experiences of relapse management as the primary outcome, using the MS relapse management scale (MSRMS) [27]. Hence EDSS/MSIS-29 was a secondary endpoint and so this small study may have been inadequately powered to identify other less marked prognostic factors. The follow-up was 6 weeks, and while the majority of patients receiving corticosteroids will have recovered, some patients may continue to improve over a longer time course [21]. Similarly, the latency from relapse-onset to treatment was somewhat prolonged (17 days) with considerable variability in the time to presentation between patients which may have masked any potential positive effect of earlier treatment.

As the study does not include an untreated control group, it is also difficult to determine the extent to which age and MSIS-29 physical scores are associated with the true treatment response to methylprednisolone as opposed to the spontaneous recovery that would occur in untreated relapses. However, this latter limitation should not detract from our study which retrospectively assesses the factors that predict responsiveness when corticosteroids are given to patient in MS relapse in the “real-world” setting of an MS relapse clinic. Indeed, there would be ethical implications about conducting a prospective study versus placebo given that corticosteroids have been shown to be clearly and repeatedly efficacious [3–7,28].

Another limitation of the study is the lack of an available EDSS and MSIS-29 physical score *prior* to relapse to enable comparative evaluation of initial relapse severity and response to treatment. Studies in which this has been done showed that patients with more severe attacks were associated with the most residual disability but were also the group that had the greatest absolute improvement in post-relapse EDSS [22]. Notably, the Cochrane review assessing the effects of intravenous versus oral corticosteroids for the treatment of MS relapses also did not incorporate pre-relapse EDSS [28].

A further larger scale but prospective single arm study would help to address the study limitations and to validate the model. Ideally, the study would assess similar variables to those in our current study but include others such as the type and duration of immunomodulatory therapy and relapse site and would incorporate pre-relapse EDSS and PRO outcomes measured before, during and after relapse. It would also be interesting to extend the length of follow-up from six weeks to perhaps three or even six months.

In conclusion, we have developed a simple scheme to predict likely response to corticosteroid treatment in individual patients experiencing an MS relapse. In this exploratory study, the most important prognostic factors for a good response, given the limitations of the study, were younger age and milder attack severity (MSIS-29 physical), though of course it is not to say that an older patient with a high MSIS-29 physical score will not necessarily benefit from corticosteroids, it is just that the anticipated chance of success is lower. This conclusion generally confirms the findings of previous studies of the importance of age and relapse severity as predictors of recovery, but is novel in extending them into a graphical representation which may be of use to clinicians when assessing individual patients with relapse in the clinic, and for the incorporation of a PRO tool as an outcome predictor.

## Supporting Information

S1 Dataset (XLSX)

S2 Dataset (XLSX)
